# Isolation and characterization of human spermatogonial stem cells

**DOI:** 10.1186/1477-7827-9-141

**Published:** 2011-10-24

**Authors:** Shixue Liu, Ziwei Tang, Tao Xiong, Wei Tang

**Affiliations:** 1 Department of Urology, the First Affiliated Hospital, Chongqing Medical University, Chongqing 400016, China; 2 West China Medical College, Sichuan University, Chengdu 610041, China

**Keywords:** spermatogonial stem cells, spermatogenesis, spermatogonia, spermatocytes, sperm cells, male infertility, andrology

## Abstract

**Background:**

To isolate and characterization of human spermatogonial stem cells from stem spermatogonium.

**Methods:**

The disassociation of spermatogonial stem cells (SSCs) were performed using enzymatic digestion of type I collagenase and trypsin. The SSCs were isolated by using Percoll density gradient centrifugation, followed by differential surface-attachment method. Octamer-4(OCT4)-positive SSC cells were further identified using immunofluorescence staining and flow cytometry technques. The purity of the human SSCs was also determined, and a co-culture system for SSCs and Sertoli cells was established.

**Results:**

The cell viability was 91.07% for the suspension of human spermatogonial stem cells dissociated using a two-step enzymatic digestion process. The cells isolated from Percoll density gradient coupled with differential surface-attachement purification were OCT4 positive, indicating the cells were human spermatogonial stem cells. The purity of isolated human spermatogonial stem cells was 86.7% as assessed by flow cytometry. The isolated SSCs were shown to form stable human spermatogonial stem cell colonies on the feeder layer of the Sertoli cells.

**Conclusions:**

The two-step enzyme digestion (by type I collagenase and trypsin) process is an economical, simple and reproducible technique for isolating human spermatogonial stem cells. With little contamination and less cell damage, this method facilitates isolated human spermatogonial stem cells to form a stable cell colony on the supporting cell layer.

## Background

The incidence of male infertility such as aspermia, oligospermia and asthenospermia of unknown origin has been increasing in recent years. Investigations on the mechanism of spermatogenesis and its influencing factors have become one of the hot topics in andrology. Spermatogenesis begins with self-renewal and differentiation of spermatogonial stem cells (SSCs). The complexity of the systemic and testicular microenvironment makes it difficult to conduct in vivo study on of SSCs. Therefore, the establishment of stable human SSCs in vitro would provide a useful stem cell model for studying the proliferation and differentiation of SSCs. The SSCs self-renewal mechanisms are tightly controlled and regulated by Sertoli cells present in the SSC niche. Specifically, the Sertoli cells secrete growth factors such as Glial cell line-derived neurotrophic factor (GDNF) that support SSCs self-renewal. Mirzapour et al [1] had isolated SSCs from human adult testes and tested their proliferation through three distinguished types of cultivation as follows, one with SSCs alone, one co-nurtured with Sertoli cells and the rest exposed to fibroblast growth factor(FGF) and human leukaemia inhibitory factor(LIF). As the result, SSCs co-nurtured with Sertoli cells proliferated with the largest number of clones. However, it was the SSCs disposed with growth factors whose diameter of clones transcended the other SSCs. In other reports, Izadyar et al [2] adopted stage-specific embryonic antigen-4(SSEA-4), CD49f and CD90 as SSC markers and demonstrated SSCs had phenotype characteristics of SSEA-4(+), CD49f(+), GPR-125(+)and c-Kit (neg/low). Additionally, Guan et al [3] testified the pluripotency of SSCs by injecting SSCs of adult mouse testis into immunodeficient mice and therefore suggested establishment of human maGSCs from testicular biopsies might allow individual cell-based therapy without the ethical and immunological problems associated with human embryonic stem.

The separation of SSCs is commonly performed by digesting testicular tissue with a multi-step enzymatic digestion process involved the use of four proteolytic enzymes. In this study, we developed a simplified two-enzyme digestion process for SSC isolation. Our results indicate that two-step enzymatic digestion is a good method to isolate testis cells.

Separation developed in our study was modified pursuant to the procedures described by previous researchers on the separation and culture of SSCs.

## Methods

### Human testis tissues

The use of human tissues was approved by the Committee on Research Ethics of Chongqing Medical University. Testis tissues were obtained from five 6-7 month old male fetuses resulted from recurrent miscarriages. The consent forms were obtained from the parents. This study has been approved by a local Ethical Committee, and written informed consent was obtained from all patients before their participation in this study. The obtained samples were assigned with a series of random 4-digit numbers to protect patient privacy.

### Reagents

DMEM/F12 medium (Hyclone Co.), Fetal bovine serum (MDgenics Co.), trypsin and collagenase I (Sigma Co.), rabbit anti-human Oct-4 first antibody, rabbit anti-human SSEA-4 first antibody, FITC fluorescein-labeled goat-anti-rabbit IgG (Beijing Biosynthesis Biotechnology Co., Ltd), and Percoll (Pharmacia Co.).

### Preparation of cell suspension from testes

Testis cells were aseptically removed within 30 min. Briefly, the testis tissues were placed in culture plates containing PBS, and the epididymis, tunica albuginea testis and fat pad were removed. The dissected testes were minced into small pieces and rinsed twice with Hank's washing solution (penicillin 200 U/ml and streptomycin 200 mg/ml). The tissue was digested with 1 g/L type I collagenase at 37°C with gentle agitation for 10 min. and then let sit for 10 min. After a brief centrifugation, the supernatant was removed and the remain pellets were added with 0.25% trypsin at a volume of 3 times that of testis tissues at 37°C for 10-15 min of gentle agitation. The digestion reaction was terminated when the contorted seminiferous tubules became soft and loose, and a great number of cells were released into solution. The disassociated cells were collected by centrifugation, and filtered through 200-mesh filtration traps. The recovered cells were cultured in DMEM/F12 culture media containing 10% fetal bovine serum. Cell viability was determined and evaluated using trypan blue.

### Percoll density gradient centrifugation

The separation and culture of SSCs was carried out as described by Bi Gang et al [4]. Briefly, Percoll was diluted to gradient concentrations of 11%, 19%, 27%, 35% and 43% with PBS. Percoll density gradient solution (2 ml at each concentration) was added to the bottom of the centrifuge tube from the highest concentration to the lowest concentration. The newly collected cell suspension was added slowly to the top layer of Percoll separation medium followed by centrifugation at 1400 rpm for 30 min. Cells at a gradient concentration ranging between 27% to and 35% Percoll were collected, washed twice with PBS, and resuspended in culture medium with a cell density adjusted to 3 × 10^6^/ml.

### Differential cell adhesion culture

The obtained cells were incubated at 37°C and 5% CO2 for 4 h, and the culture solution was pipetted gently and placed into a 25 cm culture flask. After 3 hours, the culture solution was pipetted again and the cells obtained in the culture medium were purified as putative SSCs, which were maintained at 34°C and 5% CO2.

### Isolation and culture of Sertoli cells

The cells at the gradient concentrations of 19%-27% and 35%-43% Percoll were recovered, washed twice with PBS, and then added with culture medium. The cells were seeded into 6-well culture plates and incubated at 37°C and 5% CO2.

### SSCs co-cultured with Sertoli cells

When the cultured Sertoli cells reached 90% confluency, the cells were treated with 10 g/L mitomycin C for 2-4 h, and washed 5 times with D-Hank solution. The Sertoli cells were incubated for overnight and used as feeder cells for SSCs. The isolated SSCs were inoculated on the feeder layer at a ratio of 1:1 between SSCs and Sertoli cells.

### Identification of SSCs and purity detection

Approximately 3-5 × 10^6 ^single cells were fixed with 1% paraformaldehyde for 30 min, washed 3 times with PBS, and centrifuged for collection of SSCs. After permeabilization by 0.2% Triton X100 for 10 min and 3 washes in PBS, the cells were blocked and stained with rabbit anti-human Oct-4 antibody (dilution, 1:100) at 4°C overnight after thoroughly mixing. After being washed with PBS, the fluorescent-labeled second antibody (1:100) was added for an incubation of 2 h at 4°C (in the dark), washed again with PBS, re-suspended and detected by flow cytometry (FACSAria, Becton Dickinson Co., provided by Chongqing Medical University Core Facility) with blank and negative control groups. The experiments were conducted in triplicate.

### Growth curves of SSCs

The isolated SSC cells were seeded in the 96-well plates. At 0, 24, 48, 72, 96, 120, and 144 h after plating, each well was added with 20 ul of 5 g/L tetramethyl blue tetrazolium bromide (MTT) and incubated for 4 h, Each well was added with 150 μL of DMSO and shaken for 10 min to dissolve blue particles. The absorbance A at 570 nm was detected using an enzyme-linked immunosorbent detector. The A values were expressed as mean ± SD.

### Statistical analysis

All the data were analyzed using the software SAS9.1. A p-value < 0.05 was defined as statistical significance.

## Results

### Cell viability after two-step enzyme digestion of human testicular tissues

As we isolated the SSCs using a modified protocol, we first analyzed the cell viability by counting viable cells in the cells isolated from the testes after a two-step enzymatic digestion. Our results (Table 1) demonstrate that the average rate of viable cells was 91.07%, suggesting that vast majority of the recovered cells were viable.

**Table 1:** Cell Viability after two-step emzymatic digestion

Series number	Total count (×10^6^)	Viable count (×10^6^)	Dead cell count (×10^6^)	Viability rate (%)	Dead cell rate (%)
1	4.841	4.503	0.338	93.02	6.98
2	3.782	3.365	0.417	88.97	11.03
3	6.458	6.022	0.436	93.25	6.75
4	5.568	4.985	0.583	89.53	10.47
5	3.654	3.257	0.397	89.14	10.86
Mean	4.860	4.426	0.434	91.07	8.93

### Cell adhesion and morphology

**Figure 1:**
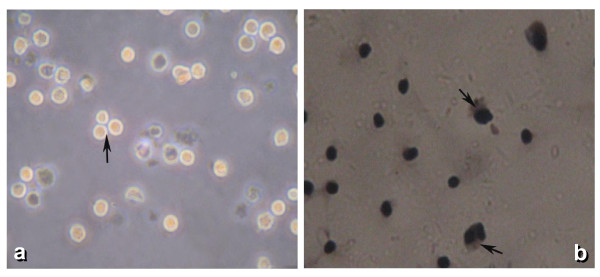
**Isolated SSCs tend to congregate and form small cell clusters**.

After culturing for 24 h, a great number of adherent SSCs were noted. Under an inverted microscope, the SSCs were seen as round or oval in shape, with a large nucleus and little cytoplasm. The isolated SSCs tended to congregate and form small cell clusters (Figure 1).

### Immunocytochemistry of stem cell marker OCT4 and SSEA-4

**Figure 2:**
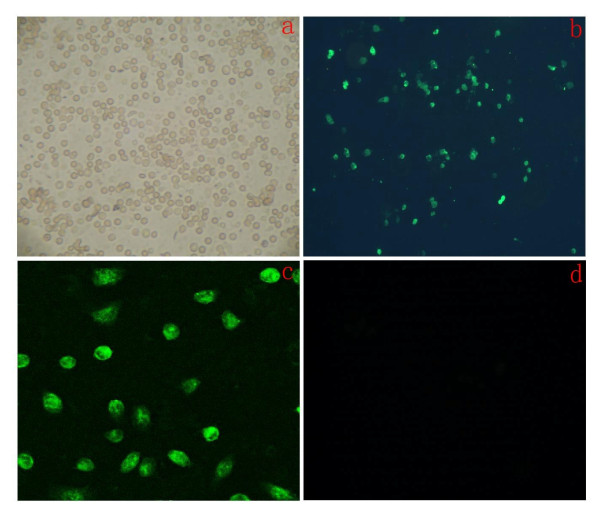
**Immunofluorecent staining of SSCs, detecte Oct-4 positive cells**. A: After Isolating, cells observed under inverted phase contrast microscope(×100)B: Show green fluorescent cells are Oct-4 positive cells observed under Immune fluorescence microscope(×100).C: Show green fluorescent cells observed under Immune fluorescence microscope(×800).D: The negative control groups of rabbit serum replace antibody, cells observed under Immune fluorescence microscope(×100).

**Figure 3:**
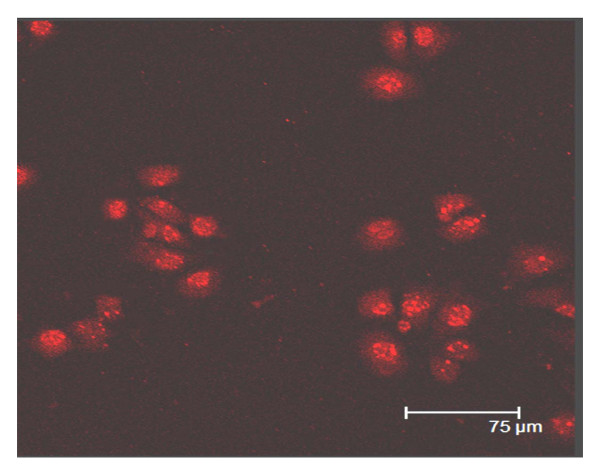
**Immunofluorecent staining of SSCs, detected SSEA-4 positive cells**. Cells were observed under immune fluorescence microscope(×400).

Using indirect immunofluorescence staining method, we found that the majority of the cells were OCT4 positive (Figure 2) and SSEA-4 positive (Figure 3). The immunostaining was observed in both cytoplasm and cell nucleus, suggesting that the isolated cells may represent SSCs.

### Co-culture of SSCs with Sertoli cells

**Figure 4:**
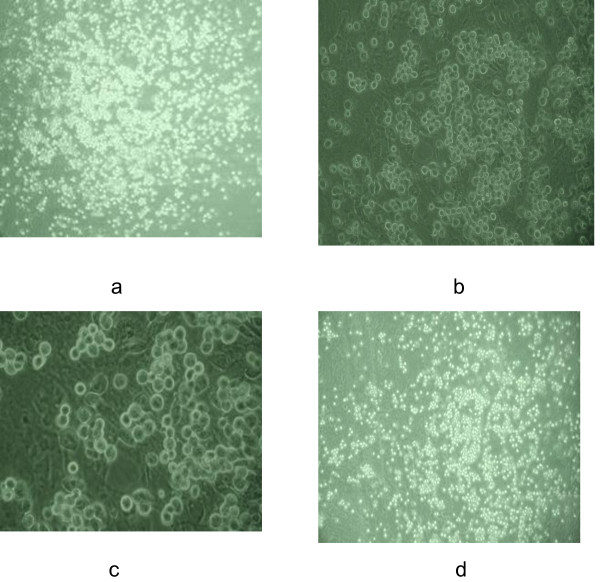
**SSCs observed under inverted phase contrast microscope at different times**. a, b, c: SSCs at 48 h of culture on the feeder layer of Sertoli cells (×100, ×200, ×400, respectively); d: SSCs at 30 days of culture on the feeder layer of Sertoli cells (×100).

Twenty-four hours after seeding, SSCs were attached to the feeder layers. At 48 h after co-culture, SSCs in the mitotic phase were apparent and the SSCs began to form cell clusters/spheres. At 72 h of culture, SSC colonies were noted (Figure 4). The medium was changed every other day for up to two weeks, and the SSC colonies were found to be stable. A great number of SSC colonies were still noted after continuous culture up to one month (Figure 4).

### Growth curve of human SSCs

**Figure 5:**
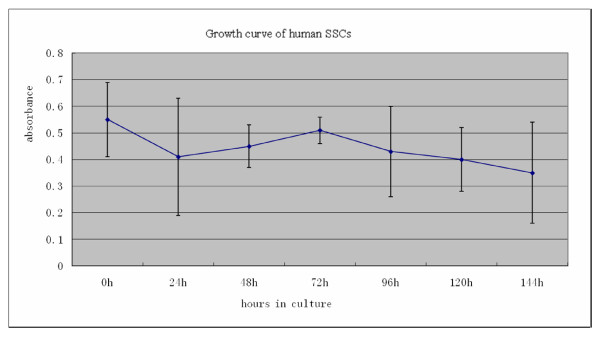
**Statistical analysis of MTT values (72 h vs. 24 h, 48 h and 96 h, P < 0.01) was performed**. The A is absorption value at a wavelength of 570 nm. Human SSCs in vitro has a value-added short period, but then gradually reduced the number of cells. The results shown are from three trials.

**Figure 6:**
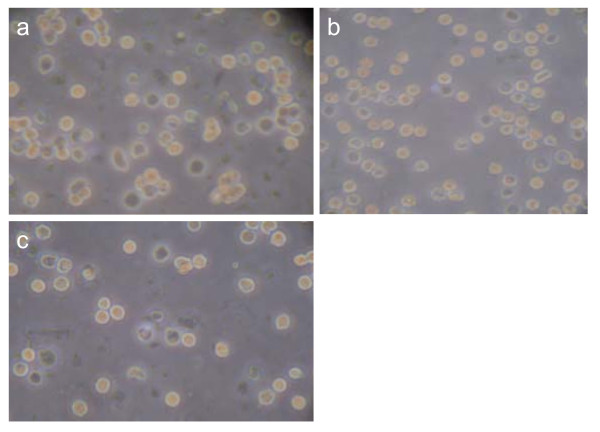
**a, b, c: SSC populations at 24 h, 72 h, and 144 h of culturing, respectively, using an inverted phase contrast microscope (×400)**. The largest number of cells in 72 h, 144 h at least.

MTT assays on SSCs were determined at 0, 24, 48, 72, 96, 120, and 144 h of culturing. The growth curve of human SSCs is shown in Figure 5. At 24 h the values of absorbance declined, rose at 48 h, and peaked at 72 h. The cell number was noted to have a gradual increase between 24 h and 72 h, followed by a decline at 96 h until reaching a minimal level at 144 h (Figure 6).

### Expression of the OCT4 stem cell marker in the isolated SSCs

**Figure 7:**
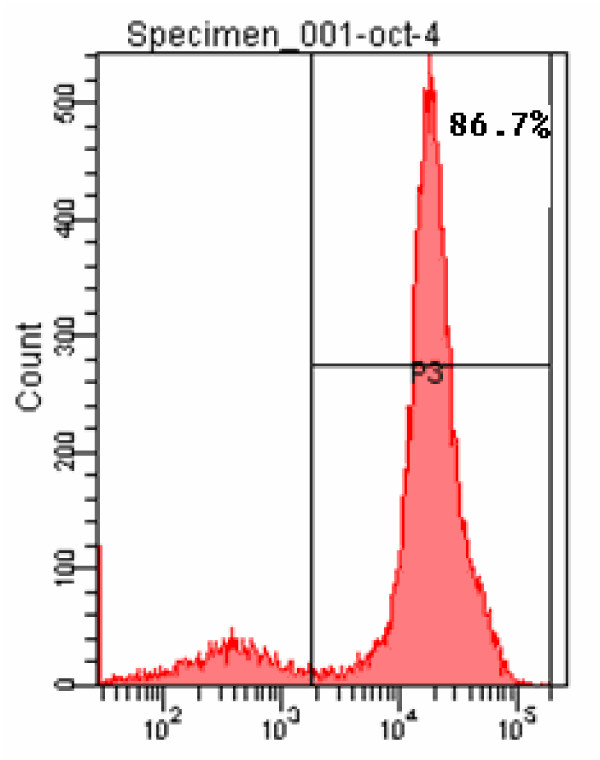
**Oct-4 positive cells detected using flow cytometry**. Two clusters of cells were segregated, of which the positive cells accounted for 86.7%.

OCT4 immunofluorescent positive cells were detected using flow cytometry with A fluorescein isothiocyanate (FITC) labeled ant-OCT4 antibody. We found that the rate of cells with positive Oct-4 labeling was 86.7% (Figure 7).

## Discussion

Stem cells are specialized cells that can differentiate into a diverse range of blast cell lines through self-renewal[5]. SSCs are a special type among stem cells, and they can transmit genetic information to the next generation[6]. Kossack et al [7] suggested the potential to derive pluripotent cells from human testis biopsies but indicated a need for novel strategies to optimize human multipotent germline stem cells (hMGSCs) culture conditions and reprogram. Singh et al [8] believed that studying the biology of SSCs will not only provide better understanding of stem cell regulation in the testis, but eventually will also be a novel target of therapy in male infertility and testicular cancer. From recent studies, cells were isolated from human testis show similar properties to embryonic stem cells [9]. Human testicular cells have great research value [9]. Spermatogenesis is a complex process that starts from the differentiation of amphiploid A-form SSCs. In seminiferous epithelium during the fetal period, spermatogenic cells at different levels are hardly seen except for SSCs and supporting cells [10]. As such, we used testicular tissue of legally induced fetuses aged 6-7 months, from which a great number of SSCs can be obtained theoretically. Currently, there are various methods for digestion of testicular tissue both at home and abroad. Multi-step, complex digestion processes, and time-consuming protocols can affect cell viability meanwhile increasing the possibility of cell contamination. Thus, a simple and feasible digestive method is crucial for preparing satisfactory uni-cell suspension. In our study, we tried the two-step enzymatic digestion multiple times and found this method to be simple and feasible, with less contamination, and by which a high rate of viable cells (91.7%) can be achieved without reducing the total cell count. These results are similar to those of Bi [4] and He et al [11], who reported to have digested human testicular tissue using 4 enzymes including collagenase, trypsin, DNA enzyme and hyaluronidase. In comparison, the method adopted in our study is more simple and feasible in operation.

Percoll is a cell separation agent with low osmolarity and no toxicity towards cells. Van Plet et al [12] reported a percentage of A spermatogonia of 76 ± 6.5% (mean ± SD) for fraction 3 (gradient range: 28%-30%) and of 80 ± 6.1% (means ± SD) for fraction 4 (gradient range: 30%-32%) when isolating A spermatogonium from the adult vitamin A-deficient rat testis using Percoll density gradient centrifugation. Lzadyar et al [13] reported a purity of about 75% when using testes from 5-month-old calves in a similar separation method. To date, there is little literature either at home or abroad on the separation of fetal testicular cells. Our experiment was conducted according to the Percoll gradient separation method described by Bi et al [4], using cells between a 27%-35% concentration gradient since SSCs were shown to be mainly distributed in this gradient range. The fast adherent velocity of the Sertoli cells makes it possible for the majority to be removed from the obtained cells after two differential adherent separations. A purity of SSCs as high as 86.7% was obtained in our study, a result is similar to that reported by Hamra [14] in which a purity of 97% was obtained for SSCs isolated from rat testicular tissue.

Oct-4 has become the marker of stem cells. It can bind to other transcription factors such as Sox and FoxD3, or to in the promoter or enhancer regions of many downstream target genes thereby positively or negatively regulating downstream gene expression in order to maintain the self-renewal of stem cells [15]. A recent study demonstrated that gonocytes, intermediate germ cells and prespermatogonia were identified during the first (7-9 wk) and second (14-19 wk) trimesters [16]. In fetal testicular tissue, Oct-4 expression is exclusively positive in SSCs but negative when expressed in type B proliferative spermatogonium. Kubota et al [17] reported that SSCs strongly expressed Oct-4 by using a green fluorescent Oct-4 antibody for labeling, thereby suggesting it to be a useful marker for SSCs. In our study, indirect immunofluorescent cell technology was used with a green fluorescent secondary antibody for labeling. The two groups of cells analyzed by flow cytometry demonstrated that 86.7% of the cells were positive for Oct-4 labeling. Under a fluorescent microscope, these cells were round and consistent with the shape of SSCs with little nonspecific staining, thereby confirming the efficacy of Oct-4 labeling for SSCs.

Little has been reported about the in vitro culture of human SSCs alone. Lim et al [18] reported SSCs derived from obstructive and non-obstructive azoospermia proliferated and maintained their characteristics for more than 12 passages (> 6 months) in vitro; moreover, the population of cells positive for the SSC-specific markers GFRalpha-1 and integrin alpha6, increased to more than 80% at passage 8. Recently, Sadri-Ardekani and associates [19] reported in vitro propagation of human prepubertal spermatogonial stem cells. Greemers et al [20] isolated type A spermatogonia from pubescent mice and cultured in a potassium-rich, serum-free medium. At days 1, 3 and 7 of culturing, the viability of the cells were shown to be 68%, 53% and 33%, respectively, indicating a downward trend in growth of type A spermatogonium. In our study, the viability of independently cultured SSCs declined at 24 h, but gradually increased at 48 h, and peaked at 72 h, after which point it declined at all remaining time intervals. These results may suggest that the decrease in viable SSCs at 24 h might be attributed to factors such as the a changed in environmental settings (from in vivo to in vitro) coupled by the impact of enzymatic digestion, in which human serum was changed to fetal calf serum etc. But at 48 h of culturing, SSCs were shown to have proliferated possibly a consequence of adaptation to the culture environment and the serum factors necessary for cell growth. After a transient proliferative phase, the number of viable SSCs began to decrease gradually possibly on account of an increasing deficiency in available growth factors such as basic fibroblast growth factor (bFGF), glial cell line-derived neurotrophic factor family receptor alpha 1(GFRα-1) and GDNF.

Currently, the conditions needed for long-term stabilization of SSCs usually consists of two categories: one is to add growth factors such as bFGF, GFRα-1 and GDNF to the media; or provide a feeder layer of cells. Kanatsu-Shinohara et al [21] established a long-term in vitro culture system of SSCs by demonstrating that a combination of growth factors, such as GDNF, induces long-term survival (over 6 months) and sustained differentiation of pluripotent SSCs. While Koruji et al [22] reported a significant increase in the number and diameter of SSC colonies by short-term co-culture with Sertoli cells. We do not use GDNF as the feeder for the SSCs because of high price of GDNF. Therefore, in our study we used human Sertoli cells as the feeder layer of SSCs and found that SSCs had a rapid adherence, marked proliferation, and formation of large numbers of colonies on the feeder layer. After 1 month of culturing there were still many colonies recognizing with the naked eye, which indicated that SSCs can form stable colonies for a prolonged period of time on Sertoli cells.

In our study, a high viability of SSCs were achieved by a two-step enzymatic digestion method with using two different enzymes, and purification by density gradient centrifugation using a discontinuous Percoll gradient and differentiated adherence technique. The results of this study pave the way for the in vitro study of differentiation, transplantation, and gene modification of human SSCs.

## List of abbreviations

SSCs: spermatogonial stem cells; FITC: fluorescein isothiocyanate; OCT4: Octamer-4; SSEA-4: stage-specific embryonic antigen-4; GDNF: glial cell line-derived neurotrophic factor; GFRα-1: glial cell line-derived neurotrophic factor family receptor alpha 1; FGF: fibroblast growth factor; LIF: leukaemia inhibitory factor; MTT: tetramethyl blue tetrazolium bromide; bFGF: basic fibroblast growth factor; hMGSCs: human multipotent germline stem cells

## Competing interests

The authors declare that they have no competing interests.

## Authors' contributions

LSX, TZW and XT carried out the isolation and characterization of human spermatogonial stem cells. LSX and TZW participated in the design of the study and performed the statistical analysis. TW conceived of the study, and participated in its design and coordination and helped to draft the manuscript. All authors read and approved the final manuscript.

## Authors' information

Wei Tang, MD, Department of Urology, the First Affiliated Hospital, Chongqing Medical University, No.1 Medical College Road, Yuzhong District, Chongqing 400016, China. Tel: 86-23-8901-1121. Fax: 86-23-6881-1487. E-mail: tangwei2060@yahoo.com.cn
